# Lost in translation: Decoding the errors in consecutive interpreting by Chinese EFL learners

**DOI:** 10.1371/journal.pone.0337758

**Published:** 2025-12-18

**Authors:** Yajin Zhuang, Liwen Chen

**Affiliations:** School of Foreign Languages/Zhejiang Gongshang University, Hangzhou, Zhejiang, China; National University of Malaysia Faculty of Education: Universiti Kebangsaan Malaysia Fakulti Pendidikan, MALAYSIA

## Abstract

Errors, as linguistic manifestations of cognitive challenges in interpreting, have attracted considerable attention in the fields of teaching, practice, and assessment. This paper examines the nature of interpreting errors among Chinese EFL (English as a Foreign Language) learners and explores their relationship with gender differences and interpreting performance, using the parallel corpus of Chinese EFL Learners-Spoken (PACCEL-S). The findings indicate the following: (1) improper speech flow was the most frequent and dense type of error, followed by grammatical errors and semantic deviations, while information default occurred least frequently. These patterns can be attributed to deficiencies in learners’ language proficiency, interpreting skills, and emotional regulation; (2) only grammatical errors showed a statistically significant correlation with gender, suggesting that gender-related differences in communication psychology may influence interpreting performance; (3) semantic deviation, information default, and improper speech flow were all significantly and negatively correlated with interpretation scores, whereas grammatical errors showed no significant correlation with these scores. These results suggest that English proficiency assessments may tolerate a certain degree of grammatical errors in interpreting tasks. Situated within the context of interpreting education, this study extends research on interpreting errors, enriches interpreting pedagogy and assessment, and deepens our understanding of the challenges faced by Chinese EFL learners.

## Introduction

Interpreting is a complex cognitive process that requires a high level of linguistic and psychological competence (e.g., [1–4]). During the process of learning to interpret, students inevitably make errors. Interpreting errors, defined as linguistic inaccuracies that occur throughout the interpreting process, serve as crucial indicators for evaluating interpreting quality and frequently used to assess language proficiency (e.g., [1,2,5]). Research on interpreting errors is therefore essential for understanding the underlying cognitive mechanisms involved in interpreting, guiding the development of interpreting pedagogy, and promoting the language skill acquisition (e.g., [6–8]).

Earlier research on interpreting errors has primarily focused on identifying their causes, often without providing a clear and consistent definition of the term “interpreting errors.” Moreover, the criteria for classifying errors remain ambiguous, with terminological inconsistencies and overlapping error categories being common (e.g., [1,9,10]). Studies examining the errors made by student interpreters are frequently limited by small sample sizes and a narrow focus on specific error types (e.g., [8,11–14]). The largest interpreting corpus in China, parallel corpus of Chinese EFL Learners-Spoken (PACCEL-S), has attracted the attention of several Chinese researchers and has been employed to investigate the interpreting errors of Chinese students, examining phenomena such as disfluency and their underlying causes [15,16]. More recently, researchers have begun to explore gender-related differences as a potential factor influencing interpreting errors, although most studies have concentrated on comparing the performance of male and female interpreters (e.g., [13,14,17,18]).

However, existing research on interpreting errors has often focused on isolated aspects, leaving the study of error characteristics relatively underexplored. Additionally, because error coding is time-consuming, many studies rely on small, non-representative samples, which may lead to biased or incomplete conclusions. Consequently, the practical implications for English language education and interpreting pedagogy in China have been somewhat limited. To address these gaps, the present study provides a comprehensive analysis of the characteristics of interpreting errors made by Chinese EFL learners in Chinese-to-English (C-E) consecutive interpreting. By utilizing the full C-E interpreting section of PACCEL-S, this study ensures both the representativeness and reliability of the data, allowing for a more accurate identification of error patterns in the interpreting process. Furthermore, by examining the interplay between interpreting errors, gender differences, and interpreting performance, the study highlights how multiple factors, such as language proficiency, interpreting skills, and emotional regulation, may influence interpreting errors. These innovations are expected to offer novel methodological and theoretical insights into the study of interpreting errors, contributing to the enhancement of interpreting education and assessment.

## Literature review

### Error Analysis (EA) theory

EA theory has received considerable scholarly attention in the field of language proficiency assessment (e.g., [14,19–21]), providing various frameworks for understanding and evaluating linguistic errors. Collectively, these frameworks reflect ongoing efforts to conceptualize the nature, classification, and implications of errors in language use. James [21] characterizes errors as manifestations of linguistic failure and proposes a comprehensive typology, including slips, mistakes, errors, and solecisms, that distinguishes between different levels of linguistic deviation. Building on this, Corder [19] differentiates between overt and covert errors, addressing both surface-level grammatical inaccuracies (overt) and deeper discourse-level issues of appropriateness or expression (covert). Hammerly [20], in turn, adds further nuance by distinguishing between distortions and faults, attributing distortions to learner behavior (either self-induced or resulting from mismanagement) and faults to inadequacies in instructional design or delivery. These diverse taxonomies highlight the conceptual complexity inherent in categorizing and analyzing linguistic errors. Despite variations in terminology and focus, a common thread in these models is the emphasis on identifying error patterns as a means of understanding the underlying causes of language breakdowns. To address the ongoing challenge of standardizing error evaluation, James [21] introduces the concept of “error gravity,” which integrates both frequency (how often an error occurs) and density (the range of error types within a given unit) of errors. This approach provides a more systematic, quantitative framework for assessing the significance of linguistic errors, thereby enhancing the analytical rigor of EA.

Although these theories have primarily been applied in second language acquisition and language assessment, their insights are increasingly relevant to interpreting studies. Research specifically applying EA to interpreting remains limited (e.g., [22]), yet EA has been recognized as a valuable tool for assessing interpreter performance and understanding the cognitive and linguistic challenges faced by interpreters. In this context, EA highlights “problem triggers” or recurrent issues encountered during interpreting tasks [1] and supports evaluations of accuracy and completeness, which are central to interpreting quality [23]. Notably, the integration of error gravity into interpreting research offers a promising methodological framework for both qualitative and quantitative analyses of interpreting discourse [22]. By combining EA theory with interpreting studies, researchers can achieve a more comprehensive and systematic assessment of interpreter performance, bridging language proficiency, cognitive processing, and communicative effectiveness. In this way, EA theory functions not merely as a diagnostic tool but as a theoretically grounded approach for advancing interpreter education, performance evaluation, and research methodology.

### Errors in interpreting

Researchers have examined interpreting errors from multiple perspectives, reflecting the complexity of defining and classifying these phenomena. Barik [9] conceptualizes interpreting errors as deviations in the target language output relative to the source language, emphasizing surface-level discrepancies. In contrast, Gile [1] frames errors as consequences of cognitive overload, linking them to working memory constraints rather than purely linguistic mismatches. Expanding on this cognitive perspective, Wehrmeyer [10] highlights the role of mental representations and processing demands, suggesting that errors arise during target language production as a result of the interpreter’s active cognitive engagement. These differing conceptualizations have given rise to varied classification systems. For example, Barik [9] categorizes errors according to the severity of deviation, omissions, additions, and substitutions, while Gile [1] distinguishes between linguistic and semantic errors, reflecting the nature of the target language output. Petite [12] adopts a process-oriented approach, identifying input-generated errors (stemming from misinterpretation of the source) and output-generated errors (resulting from formulation issues in the target language). Gerver [24] further differentiated errors by their source, distinguishing passive errors, caused by external disruptions such as noise, from active errors, rooted in internal factors such as physiological or socio-cultural conditions. Collectively, these diverse definitions and typologies underscore the lack of consensus in the field and highlight the need for a more integrated framework. The current fragmentation in terminology and focus points to a critical gap that further research must address to clarify the nature of interpreting errors and improve their application in both assessment and pedagogy.

### Research on interpreting errors

Interpreting activities share fundamental similarities with oral speech production, particularly in their reliance on real-time verbal expression. However, the cognitive mechanisms underlying interpreting are considerable more complex. Unlike typical speech, interpreting requires the immediate and accurate transfer of a message from a source language to a target language, often in a single, uninterrupted pass, placing substantially greater demands on cognitive resources [2]. The “tightrope hypothesis” illustrates this heightened strain, suggesting that the dual demands of managing attention and cognitive load in interpreting approach a critical threshold of saturation, thereby increasing the likelihood of errors [1]. Consequently, the psychological mechanisms underlying interpreting errors differ from those involved in regular speech production, reflecting the elevated cognitive demands of the task.

A growing body of research has examined the nature and sources of interpreting errors, particularly among professional interpreters. Several studies provide insight into different dimensions of this issue. For example, Bendazzoli, Sandrelli, and Russo [25], using data from the European Parliament Interpreting Corpus (EPIC), found that phonological slips, such as mispronunciations or incomplete articulations, occurred more frequently when the source and target languages were typologically similar. This suggests that increased linguistic proximity may heighten the risk of certain error types, possibly due to interference or competition between closely related lexical items. Complementing these findings, Chang [26] analyzed 146 C-E interpreting test recordings and identified patterns among lower-scoring performances, including slower speech rates, greater disfluency, and more comprehension errors. Interestingly, rates of grammatical and lexical errors were relatively consistent across both high- and low-scoring groups, indicating that fluency and comprehension may be more sensitive indicators of interpreting performance under cognitive strain. Wehrmeyer [10] adds another perspective by examining errors in sign language interpreting. Across ten simultaneous interpreting sessions conducted by two professionals, pronunciation and conceptualization errors were most common, whereas comprehension errors were relatively rare. Notably, no correlation was found between error frequency and session duration, suggesting that cognitive overload plays a more significant role than fatigue or time-on-task alone. Collectively, these studies highlight the multifaceted nature of interpreting errors and point toward cognitive overload as a unifying explanatory factor. However, inconsistencies, arising from variations in sample size, language pairs, and interpreting modalities, complicate efforts to draw generalizable conclusions, underscoring the need for further empirical research using more controlled conditions and diverse datasets to better understand the cognitive underpinnings of interpreting performance.

Moreover, empirical research on interpreting errors often relies on small sample sizes to investigate the underlying causes of errors and to assess the applicability of EA theory in evaluating interpreting quality. For example, Wang [22], analyzing data from 14 contestants in the finals of the 3rd CTPC Cup All China Interpreting Contest, identified numbers, nouns (or names), and logical relationships as the three main problem triggers in simultaneous interpreting, with varying significance across both C-E and English-to-Chinese (E-C) directions. Similarly, Lu et al. [8] examined the types and causes of errors produced by four English majors during classroom interpreting exercises, finding that comprehension errors were most frequent and occurred more often in the E-C direction than in C-E interpretation. Additionally, grammatical errors and disfluencies were more prevalent in the C-E direction than in the reverse. Other studies have also suggested that external factors, including language proficiency, gender-related differences, and the ability to process the source language, may contribute to variations in interpreting errors and subsequently influence interpreting performance (e.g., [4,15,27,28]). However, these studies are often limited by inconsistent error classification criteria and a tendency to focus on a single error type, restricting the comprehensiveness and generalizability of their findings.

Previous research has identified notable gender-based differences in interpreting performance, particularly in the types and frequencies of interpreting errors (e.g., [18]). Studies suggest that female interpreters tend to exhibit greater accuracy in content fidelity, appropriate terminology usage, and grammatical correctness, whereas male interpreters often demonstrate strengths in lexical diversity and overall fluency (e.g., [14,17,29]). These findings highlight gender as a potentially influential factor in interpreting performance, contributing to broader discussions in both interpreting studies and English language teaching research. However, despite growing interest in this area, little is known about how gender-related patterns manifest specifically within the context of English language education in China. In particular, the extent to which gender influences the nature and frequency of interpreting errors among Chinese EFL learners remains underexplored. Addressing this gap is central to the present study, which aims to examine how gender may shape error types and interpreting outcomes in this educational context.

In parallel, the study of interpreting errors, often conceptualized as “an unsuccessful bit of language” [21], has played a vital role in enhancing interpreter training by improving learners’ self-monitoring strategies and overall language proficiency. While existing literature identifies a range of factors contributing to interpreting errors, including cognitive overload, time constraints, limited language competence, interpreter fatigue, and task complexity (e.g., [1,10,26]), these insights have primarily emerged from professional interpreting contexts or general language learning environments. There remains limited understanding of how these factors interact within the experiences of Chinese EFL learners specifically. Furthermore, the interplay between interpreting errors, gender differences, and performance outcomes has not been systematically examined. Investigating this intersection offers valuable implications for both interpreting pedagogy and the development of gender-responsive approaches to English language education.

Building on insights from the existing literature and situated within the context of English language education in China, the present study examines the characteristics of interpreting errors among Chinese EFL learners, with a particular focus on gender-related differences and their relationship to interpreting performance. By addressing a gap in current research, this study aims to enhance understanding of how learner-specific variables influence interpreting outcomes in educational settings. Specifically, the study investigates the following research questions:

1. What are the distinctive characteristics of interpreting errors made by Chinese EFL learners in terms of error types, frequency, and density in C-E interpreting tasks?
2. To what extent do gender-related differences influence the interpreting errors produced by Chinese EFL learners?
3. What is the relationship between the interpreting errors made by Chinese EFL learners and their overall interpreting performance?

## Methodology

### Data source

EA is a data-driven methodology that primarily relies on evidence from learners’ output, self-reported data, or information obtained through verbal protocols (e.g., [21,30]). This study utilizes data from the publicly available PACCEL-S, maintained by Beijing Foreign Studies University. The original data collection procedures were conducted with ethical approval from the corpus developers, and the corpus’s terms of use explicitly permit secondary analysis. All analyzed data are fully anonymized aggregates containing no identifiable personal information, in compliance with international ethical standards (e.g., Declaration of Helsinki, Paragraph 23, on the use of public data). The present study was conducted in accordance with the recommendations of the Ethics Committee of Zhejiang Gongshang University.

The data for this study were drawn from the C-E interpreting section of PACCEL-S, which comprises recordings of interpreting tests completed by Chinese fourth-year English majors during the oral examination of the Test for English Majors-Band 8 (TEM-8) between 2003 and 2007. The corpus is organized into two thematic domains, economics and culture, providing a valuable resource for assessing the language proficiency and learning progress of Chinese EFL learners, particularly those at the beginner level of interpreting [31].

Unlike interpreting tasks in real-world scenarios, the interpreting section of the TEM-8 oral exam is a simulated task conducted under controlled test conditions. It serves as a formal assessment of learners’ English proficiency and provides critical data for the training and selection of interpreting talent [31]. To ensure the representativeness and comprehensiveness of the corpus, the present study utilizes the full dataset from the C-E interpreting section of PACCEL-S, excluding samples that contain only header metadata without transcription text, as well as those lacking gender or performance information. This process yielded 918 valid samples, comprising 166,454 word tokens and 5,700 word types

The TEM-8 oral exam employs a stringent scoring system, with the final score reflecting the examinee’s actual interpreting proficiency (e.g., [15,31,32]). Based on these evaluation criteria, a comprehensive Chinese learner interpreting error feature observation corpus has been established, ensuring a high level of data reliability. This study follows the two-stage EA framework proposed by McDowell and Liardét [33], which involves: (1) error recognition and reconstruction, and (2) error classification and quantification. Ethical clearance for the study was obtained from the oversight committee at the first author’s institution prior to data analysis.

The corpus for the present study was manually coded according to the variable descriptions outlined above, with the coding process undergoing three rounds of comprehensive proofreading to ensure accuracy. The coded data were subsequently imported into SPSS 27 for statistical analysis, focusing on key variables such as the types, frequency, and density of interpreting errors, along with other relevant metrics. Using SPSS’s advanced quantitative analysis capabilities, both parametric tests and correlation analyses were conducted.

### Variable settings

Although scholars offer varying interpretations regarding the nature of interpreting errors, most classifications generally distinguish between errors related to content and those associated with the form of target language production. Content in target language expression primarily concerns the accuracy, fidelity, and coherence of the conveyed message, whereas form pertains to aspects of language use, vocal expression, and non-verbal expression. Language use involves four key quality parameters: correct pronunciation, grammatical accuracy, naturalness, and appropriateness. Verbal expression encompasses phonetic features, intonation, voice quality, volume, and fluency, while non-verbal expression includes eye contact, facial expressions, gestures, and posture (e.g., [1,2,6,34]). Moreover, other scholars maintain that interpreting quality should be evaluated based on both content and form, with content referring to the accuracy and completeness of meaning and form relating to the correctness and fluency of language [35–38].

Consistent with the nature of interpreting tasks, interpreting errors can be classified into content errors and form errors. Content errors primarily involve inaccuracies in the conveyed information, whereas form errors encompass linguistic mistakes, phonological discrepancies, and issues related to speech flow. Drawing on the textual features of the C-E interpreting corpus of PACCEL-S, the present study investigates the characteristics of interpreting errors made by Chinese EFL learners across four key dimensions^1^: Semantic Deviation, Information Default, Grammatical Error, and Improper Speech Flow (see Table 1). The variable descriptions provided in Table 1 serve as the principal observational framework for this study.

**Table 1:** Description of four types of interpreting errors.

Error type	Error description	Coding	Error description
Semantic Deviation (SD)	Errors arising from a misinterpretation of the source language or the inappropriate expression in the target language, resulting in a discrepancy in meaning between the source and target texts. E.g., mistranslation, unwarranted additions, over-explication, etc.	[SD]	SL: çºªå¿µæ´"åŠ¨è¡¨è3/43/4äº†å¯¹åŽ†å#143;²çš"å°Šé‡#141; *(The commemorative activity expressed respect for history.)* TL: The summary activity[SD] show[GE] the respect to the history...
Information Default (ID)	Errors occurring during the conversion of source information into the target language, wherein essential information is either partially conveyed or omitted altogether, leading to significant gaps in meaning. E.g., major information omissions, insufficient explication, etc.	[ID]	SL: æˆ’ä"¬å¸Œæœ›å€Ÿé‰´å’Œå­¦ä¹ å›1/2å¤-çš"å...ˆè¿›æŠ€æœ¯å’Œç"#143;éªŒ *(We hope to draw on and learn advanced technologies and experiences from abroad.)* TL: We hope we can provide a good opportunity to[GE] the manufacturers and producers to learn advanced[ID] experience from the foreign countries.
Grammatical Error (GM)	Errors in target language expression that violate established English grammatical conventions, including but not limited to errors in tense usage, subject-verb agreement, incorrect inflection of verbs or nouns, and syntactic structures that deviate from standard grammatical norms.	[GE]	TL: At the same time, we provide[GE] opportunity for the, er[IF], music, for the music-lover...
Improper Speech Flow (IF)	Disfluencies in target language output, encompassing interruptions in speech flow such as unnecessary (silent) pauses, redundant repetitions, and self-repairs.	[IF]	TL: The purpose of this meet, exhibition is to strengthen the international cooperation er[IF] uhm[IF] in this field.

**Semantic Deviation:** Semantic Deviation refers to instances of mistranslation that result in a divergence of meaning between the source and target languages (e.g., [1,2,9]). This category encompasses errors such as misinterpretations or unwarranted additions, where the message conveyed in the target language deviates from the intended meaning in the source language. Such discrepancies may involve both semantic distortions and unintended shifts in meaning, leading to a misalignment between the two languages.

**Information Default:** Information Default denotes the omission of essential elements from the source language, resulting in an incomplete or distorted representation of the original message in the target language. In the present study, this category specifically addresses the unintentional omission of critical information during the interpreting process, while excluding strategic omissions made for efficiency purposes (e.g., abbreviation, condensation, or compression) (e.g., [1,2,9]). Unlike intentional strategies designed to enhance efficiency, the omissions analyzed in this study refer solely to unplanned exclusions of important content, and strategic omissions are therefore excluded from both analysis and discussion.

**Grammatical Error:** Grammatical Errors refer to instances in which target language expressions deviate from established English grammatical norms, including errors such as tense inconsistencies, subject-verb disagreement, incorrect verb or noun inflections, and syntactic anomalies. While some scholars suggest using frequency as a basis for assessing the severity of grammatical errors, others advocate categorizing errors according to varying levels of severity (e.g., [19,21]). However, there is no consensus on standardized criteria for evaluating their severity. In the present study, we adopt an objective approach, assessing grammatical errors solely based on deviations from established English grammar norms, thereby ensuring a consistent and measurable framework for analysis.

**Improper Speech Flow:** Improper Speech Flow pertains to disruptions in the fluency of target language output, including phenomena such as pauses, repetitions, and self-repairs, which serve as key indicators of speech fluency (e.g., [1,2,34]). Interpreting fluency encompasses both utterance fluency and perceived fluency: the former refers to the narrow concept of fluency within the speech itself, while the latter reflects the listener’s subjective perception of the target language discourse [39]. The present study specifically examines the impact of discourse fluency on interpreting performance. Pauses, repetitions, and corrections are among the primary indicators of discourse fluency in interpreting (e.g., [1,2]), and the term “improper speech flow” is used to distinguish this category from other forms of disfluency.

## Results

### Nature of C-E interpreting errors

**Fig 1:**
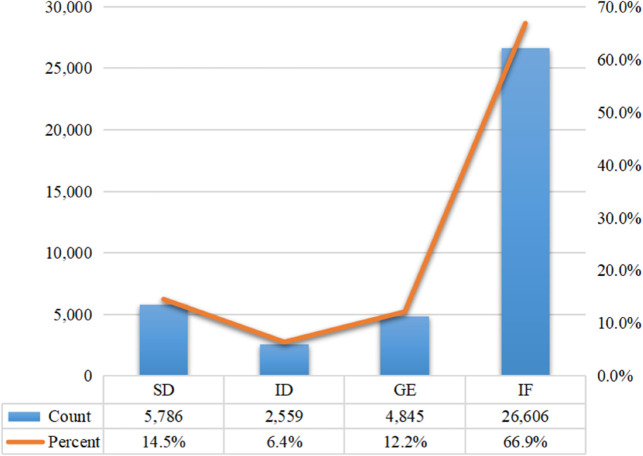
Statistics of error types in C-E interpreting by Chinese EFL learners.

The analysis of interpreting errors in the present study focuses on two main aspects: error types and error severity, specifically examining four categories of errors: semantic deviation, information default, grammatical error, and improper speech flow. According to the corpus statistics, the distribution of each error type is presented in Fig 1. As shown, improper speech flow was the most prevalent error, occurring 26,606 times and accounting for 66.9% of all errors. This was followed by semantic deviation (5,786 occurrences, 14.5%), grammatical errors (4,845 occurrences, 12.2%), and information default, which was the least frequent (2,559 occurrences, 6.4%).

The quantification of error severity in the present study is based on two primary metrics: error frequency and error density. Error frequency refers to the number of occurrences of a specific error type within a single interpreting discourse, whereas error density indicates the proportion of errors relative to the total number of words in the discourse [21]. Based on the token counts and statistical data for each error type in the corpus, the relevant metrics for error severity in C-E interpreting are summarized in Table 2. As the error frequency data correspond to the previously reported error counts, these results are consistent with those presented earlier and will not be reiterated here. As shown in Table 2, improper speech flow exhibits the highest density, occurring at a rate of 16 instances per 100 words, followed by semantic deviation (3.5 per 100 words), grammatical errors (2.9 per 100 words), information default, which is the least dense at 1.5 per 100 words.

**Table 2:** Severity statistics of C-E interpreting errors by Chinese EFL learners.

Error type	Semantic Deviation	Information Default	Grammatical Error	Improper Speech Flow
*Frequency*	5,786	2,559	4,845	26,606
*Density*	3.5%	1.5%	2.9%	16.0%

### The relationship between C-E interpreting errors and gender difference

To investigate potential gender-related differences in C-E interpreting errors, an independent samples *t*-test was conducted. The results are summarized in Table 3.

**Table 3:** Independent samples *t*-test for the correlation between C-E interpreting errors and gender difference.

Error type	Gender	N	Mean	t	p (2-tailed)	MD	95% CI		Cohen’s d
							Lower bound	Upper bound	
Semantic Deviation	M	133	6.8421	1.754	0.080	0.64593	-0.07699	1.36885	0.255
	F	785	6.1962				-0.1151	1.40696	
Information Default	M	133	3.1053	1.419	0.158	0.37278	-0.0794	0.82496	0.222
	F	785	2.7325				-0.14588	0.89144	
Grammatical Error	M	133	6.1278	3.570	0.000**	1.00425	0.45218	1.55633	0.440
	F	785	5.1236				0.42794	1.58056	
Improper Speech Flow	M	133	26.1429	-1.910	0.056	-3.32084	-6.7336	0.09193	-0.617
	F	785	29.4637				-6.77222	0.13055	

Note: MD = Mean Difference; CI = Confidence Interval; *p* < .01 (**) indicates statistical significance; Cohen’s *d* denotes effect size.

Based on Table 3, the major findings were as follows:

**1.Mean differences and effect sizes:** Across most error types, gender-based differences in mean scores were observed. Male participants exhibited higher mean frequencies of semantic deviations, information defaults, and grammatical errors, suggesting potential challenges in source-text comprehension and reformulation. In contrast, female participants showed a higher mean score for improper speech flow, which may reflect difficulties in maintaining fluency under cognitive pressure. The greater incidence of grammatical, semantic, and omission errors among males may indicate challenges in accurately analyzing and reformulating source content, likely arising from excessive cognitive load. When cognitive demands exceed working memory capacity, performance declines, particularly in linguistically or conceptually complex tasks such as interpreting.

Importantly, Cohen’s *d* values provide insight into the magnitude of the observed gender differences. Specifically: (1) Grammatical Errors (*d* = 0.440, moderate effect) support earlier hypotheses regarding the challenges male participants face in allocating cognitive resources to analysis; (2) Improper Speech Flow (*d* = -0.617, moderate-to-large effect, favoring males) suggests that male interpreters may exercise superior control over production efforts under cognitive pressure; and (3) Semantic Deviation and Information Default exhibit smaller effect sizes (*d* = 0.255 and *d* = 0.222, respectively), indicating modest gender differences in these areas. Although some comparisons did not reach conventional levels of statistical significance, the effect sizes highlight the practical relevance of the observed differences. The gradient of effect sizes across error types further supports the presence of gender-based variation in cognitive resource allocation during interpreting. Specifically, disfluencies appear more closely linked to differences in the management of coordination effort, whereas grammatical and semantic errors primarily reflect disparities in the distribution of analysis-related cognitive resources [1].

**2. Statistical significance and confidence intervals:** Grammatical Error was the only error type to exhibit a statistically significant gender difference (*t* = 3.570, *p* < .001), with a moderate effect size (*d* = 0.440) and 95% confidence interval that did not include zero (CI: [0.45218, 1.55633]). In contrast, Semantic Deviation (*p* = 0.080), Information Default (*p* = 0.158), and Improper Speech Flow (*p* = 0.056) were not statistically significant, with confidence intervals that included zero, indicating no robust evidence of gender differences in these categories at the 0.05 level. Nevertheless, the moderate-to-large effect size for Improper Speech Flow (*d* = -0.617) suggests a substantive trend that warrants further investigation in future studies.

**3. Pearson correlation with gender:** To further examine the relationship between grammatical errors and gender, a Pearson correlation analysis was conducted. A significant negative correlation was found at the 0.010 level (two-tailed) (*r* = -0.117, *p* = 0.000 < 0.010). Although small, this significant correlation indicates that as gender shifts from male to female, the frequency of grammatical errors tends to decrease. This finding corroborates the earlier *t*-test results and suggests a modest association between gender and syntactic accuracy. The result also implies that male participants may experience higher cognitive strain when managing syntactic accuracy alongside the other demands of interpreting. Alternatively, differences in grammatical accuracy may reflect variations in access to or engagement with language-rich environments, which are critical for internalizing syntactic rules through natural input [40].

### The relationship between C-E interpreting errors and interpreting performance

In the present study, interpreting performance was categorized into three levels, Level 1 (high scores), Level 2 (medium scores), and Level 3 (low scores), based on the grade distribution within the sample [31]. To determine whether significant differences existed among these performance levels, a Scheffé test was conducted (see Table 4).

**Table 4:** Scheffé test for three groups in C-E interpreting performance.

Sources	Sum of squares	df	Mean square	F	Significance
*BetweenGroups*	113,421.037	2	56,710.519	860.903	< 0.001
*WithinGroups*	60,274.093	915	65.873		
*Total*	173,695.13	917			

The results revealed statistically significant differences between groups (*p* < 0.001), warranting further analysis. Subsequently, a Pearson correlation analysis was performed to examine the relationship between error types and interpreting performance levels. The findings, summarized in Table 5, are as follows:

**Table 5:** Correlation analysis between C-E interpreting errors and scores.

		Score	Semantic Deviation	Information Default	Grammatical Error	Improper Speech Flow
Score	Pearson correlation	1.000	-0.364**	-0.483**	0.002	-0.225**
	Significance (2-tailed)		0.000	0.000	0.957	0.000
Semantic Deviation	Pearson correlation	-0.364**	1.000	0.324**	0.028	0.192**
	Significance (2-tailed)	0.000		0.000	0.391	0.000
Information Default	Pearson correlation	-0.483**	0.324**	1.000	-0.080	0.212**
	Significance (2-tailed)	0.000	0.000		0.016	0.000
Grammatical Error	Pearson correlation	0.002	0.028	-0.080	1.000	0.004
	Significance (2-tailed)	0.957	0.391	0.016		0.913
Improper Speech Flow	Pearson correlation	-0.225**	0.192**	0.212**	0.004	1.000
	Significance (2-tailed)	0.000	0.000	0.000	0.913	

Note: * and ** respectively refer to *p*-value smaller than .050 and .010.

**1. Correlation strength:** Three error types, semantic deviation (*r* = -0.364), information default (*r* = -0.483), and improper speech flow (*r* = -0.225), demonstrated moderate negative linear correlations with interpreting performance, with correlation coefficients ranging between 0.300 and 0.500. This indicates that as the frequency of these errors increases, interpreting performance tends to decrease. Among them, the strongest negative correlation was observed for information default, followed by semantic deviation and improper speech flow. The strong association between information default and lower performance suggests that cognitive overload, particularly in memory and comprehension, may impede the retention and delivery of essential information. Likewise, semantic deviation may reflect difficulties during the “listening and analysis” phase, where interpreters struggle to accurately comprehend or reformulate the intended meaning due to processing bottlenecks. In contrast, grammatical errors exhibited a negligible correlation with performance (*r* = 0.002), which was not statistically significant (*p* = 0.957). This finding suggests that surface-level grammatical accuracy may not be a major determinant of overall interpreting quality, especially in high-pressure, real-time interpreting contexts.

**2. Statistical significance:** The *p*-values for semantic deviation, information default, and improper speech flow were all below the 0.010 threshold, confirming the statistical significance of their negative correlations with interpreting performance. In contrast, the lack of statistical significance for grammatical errors further highlights their limited role in influencing performance outcomes in this context. These findings support the existence of a “hierarchy of effects” in interpreting errors, in which semantic completeness (information default) and conceptual accuracy (semantic deviation) exert a substantially greater impact on communicative effectiveness than linguistic formality (grammatical errors). Moreover, the three error types that showed significant correlations correspond closely to the key cognitive phases in Gile’s model: information default aligns with memory effort, semantic deviation with listening and analysis effort, and improper speech flow with production effort [1].

## Discussion

Drawing on the C-E interpreting corpus PACCEL-S, this study investigates the characteristics of interpreting errors committed by Chinese EFL learners in C-E interpreting exams. It further examines the interrelationships among these errors, gender differences, and overall interpreting performance. The discussion section revisits the research questions and provides a critical analysis of the findings, situating them within the broader context of interpreting studies and second language acquisition research.

First, the study analyzed the nature and distribution of interpreting errors produced by Chinese EFL learners. The results indicated that the overall density of semantic deviation, grammatical errors, and information default was relatively low, suggesting that participants generally demonstrated a high degree of accuracy and completeness in conveying source language content. These findings imply that the interpreting component of the TEM-8 oral examination, designed to assess learners’ language proficiency, represents a moderate level of difficulty and is appropriately calibrated to evaluate their language competence. Nonetheless, improper speech flow emerged as the most frequently occurring error type. Given that all participants were fourth-year English majors, several factors may account for the observed error patterns:

**1. Insufficient Language Proficiency:** Prior research highlights the critical role of language proficiency during the early stages of interpreting training (e.g., [10,26,41–43]). The interpreting section of the TEM-8, primarily designed to assess linguistic ability, places considerable demands on students’ English proficiency [32]. At this stage of interpreter education, learners’ interpreting performance largely depends on their command of the target language. The presence of semantic deviations and grammatical errors suggests that some students still face challenges in using English effectively to achieve accurate message transfer and produce syntactically coherent output.

**2. Limited Interpreting Skills:** A lack of formal interpreting training and limited practical experience can hinder learners’ ability to effectively monitor and manage their output. Interpreting requires the rapid retrieval and application of appropriate lexical and syntactic forms under time constraints, a process that demands a high level of automaticity and strategic competence (e.g., [1,2,42]). Learners without sufficient training often struggle to produce smooth, fluent interpretations. When difficulties arise, they may lack the problem-solving strategies necessary to maintain communicative coherence (e.g., [2,44]), resulting in speech disruptions that can subsequently lead to semantic inaccuracies or the omission of critical information.

**3. Insufficient Emotional Regulation:** Emotional factors play a significant role in interpreting performance. Many students struggle to manage emotional stressors during interpreting tasks, which can undermine cognitive processing and communicative effectiveness. Factors such as anxiety, low self-efficacy, negative affect, or inattentiveness can increase stress levels, impeding real-time information processing and compromising output quality (e.g., [1,4,28]). Additionally, limited interpreting experience may contribute to cognitive disorganization, exacerbating performance anxiety and creating a self-reinforcing cycle of errors (e.g., [12,44,45]).

These findings align with Gile’s Effort Model of consecutive interpreting, which conceptualizes interpreting as a cognitively demanding activity comprising four concurrent efforts: listening and analysis, note-taking, short-term memory operations, and coordination [1]. The high incidence of disfluency-related errors, characterized by pauses, repetitions, and self-repairs, suggests that learners may experience cognitive overload, particularly in managing the coordination effort that integrates multiple cognitive processes. Such disfluencies likely indicate a breakdown in coordination, where interpreters struggle to balance comprehension and reformulation under time constraints. Furthermore, the prominence of fluency-related disruptions may reflect elevated intrinsic cognitive load, stemming from the inherent complexity of the interpreting task, as well as extraneous cognitive load [40], potentially resulting from underdeveloped language processing strategies or insufficient training. In contrast, the relatively lower frequencies of information default and grammatical errors may indicate a stronger grasp of fundamental linguistic structures, though not necessarily the real-time fluency required in interpreting. Collectively, these findings underscore the need for pedagogical interventions that extend beyond language proficiency development to include targeted training in cognitive load management. Specifically, instructional approaches that focus on improving coordination, automatization, and strategic processing may better prepare learners for the demands of real-time interpreting.

Second, this study examined the relationship between gender and interpreting errors among Chinese EFL learners. The analysis revealed that, with the exception of grammatical errors, there were no statistically significant gender differences in the incidence of semantic deviation, information default, or improper speech flow. Overall, male participants demonstrated lower accuracy in their interpretations compared to their female counterparts, although their fluency was notably higher. While gender differences in interpreting performance were limited in terms of statistical significance, the observed disparity in grammatical error rates, combined with moderate effect sizes, suggests the presence of meaningful underlying variation. These differences may be attributed to several factors: (1) cognitive factors, such as differences in working memory capacity and processing efficiency; and (2) input-related factors, including variations in exposure to language-rich environments, which are essential for developing grammatical competence.

These findings are consistent with previous research (e.g., [14,17,29,46]) and may reflect gender-based differences in communicative strategies. Specifically, male learners tend to prioritize the transmission of communicative intent over precise lexical and syntactic choices, whereas female learners often emphasize linguistic accuracy and appropriateness (e.g., [15,17,29]). This distinction supports the observed gender-related variation in grammatical accuracy: male interpreters may accept compromises in grammatical correctness to maintain fluency, while female interpreters are more likely to engage in heightened self-monitoring to ensure linguistic accuracy. However, this heightened monitoring can sometimes result in overcorrections or repetitions that negatively affect fluency. From a pedagogical perspective, male interpreting students may benefit from targeted training focused on the analysis phase, particularly in enhancing source language comprehension and grammatical precision. Conversely, female students may benefit from developing strategies for more effective coordination effort management, enabling them to sustain fluency under cognitive load.

These findings provide deeper insight into Gile’s Effort Model. When total cognitive demand approaches or exceeds working memory capacity, interpreters may adopt gender-differentiated strategies for allocating cognitive resources, resulting in systematic differences in error patterns [1]. In this context, statistical significance primarily reflects the degree of inferential certainty within the sample, whereas effect sizes and confidence intervals offer more substantive insights into the nature and magnitude of these differences. Together, these measures provide a nuanced evidential foundation for refining cognitive models of interpreting, particularly regarding individual and gender-based variability in resource allocation.

Third, this study examined the relationship between interpreting errors and overall interpreting performance among Chinese EFL learners. The findings revealed that semantic deviation, information default, and improper speech flow all had significant negative impacts on interpreting performance, whereas grammatical errors showed no statistically significant correlation with interpreting scores. It is widely acknowledged that the accuracy and comprehensiveness of meaning transmission are fundamental to effective interpreting (e.g., [3,35,37,47]). Accuracy refers to the correctness of the conveyed content, while comprehensiveness pertains to the completeness of the transmitted information. Accordingly, both information default and semantic deviation were found to significantly affect the overall quality of interpretation, as these error types compromise the core objective of faithfully reproducing the source message. Fluency, in contrast, encompasses the coherence, timing, and rhythmic delivery of the target language, which are essential for ensuring the intelligibility and naturalness of interpretation (e.g., [25,26]). Therefore, improper speech flow can substantially diminish the perceived quality and effectiveness of interpretation (e.g., [8,12,15,22]). These findings further support Krashen’s Input Hypothesis, which posits that language acquisition, and, by extension, language fluency, develops more effectively through exposure to comprehensible input rather than through an explicit focus on grammatical form [40]. In the context of interpreting, minor grammatical inaccuracies may not hinder effective communication, provided that the intended meaning is preserved and clearly reformulated.

According to the scoring criteria of the TEM-8, the “Grammar and Vocabulary” section permits a certain degree of grammatical errors and lexical imprecision. These variations are incorporated within the broader performance descriptors (“Excellent,” “Good,” “Pass,” and “Fail”) and are not treated as determinative of overall success [31]. Some scholars further argue that while linguistic accuracy is a relevant dimension of performance, its weight in holistic interpreting assessment should be moderate, with an optimal influence range of approximately 30%-50% of the total evaluation (e.g., [3,32,48]). The absence of a significant correlation between grammatical errors and interpreting performance, therefore reflects the TEM-8’s allowance for a degree of grammatical imperfection. It also underscores the importance of a holistic approach to evaluating interpreting competence, one that prioritizes the effectiveness of meaning transmission over strict formal accuracy. This consideration is particularly relevant under conditions of cognitive overload, where interpreters must often allocate limited cognitive resources toward preserving meaning rather than maintaining grammatical precision.

These findings provide empirical support for the development of interpreting assessment frameworks that prioritize communicative effectiveness as the central criterion. They also emphasize the importance of focusing on the most impactful error types, semantic deviation, information default, and improper speech flow, in interpreter training programs, as these errors have the greatest influence on communicative success and audience comprehension.

## Conclusion

Drawing on the C-E interpreting corpus of PACCEL-S, the present study provides a comprehensive EA of the interpreting performance of Chinese EFL learners. The findings indicate that among the four identified error categories, improper speech flow was the most frequent and dense, followed by grammatical errors and semantic deviation, with information default being the least prevalent. Contributing factors include limited language proficiency, insufficient training in interpreting techniques, and underdeveloped emotional regulation skills. These results underscore the complex and multidimensional nature of interpreting challenges faced by EFL learners and highlight the need for more targeted, practice-oriented instructional approaches.

Further analysis revealed that, while semantic deviation, information default, and improper speech flow showed no significant gender-based differences, grammatical errors were statistically correlated with gender, potentially reflecting underlying psychological and communicative differences. Additionally, semantic deviation, information default, and improper speech flow were found to have a substantial negative impact on overall interpreting performance. In contrast, grammatical errors did not significantly correlate with interpreting scores, a finding consistent with the TEM-8 oral exam scoring rubric, which allows a certain degree of grammatical imperfection when assessing interpreting competence [31].

Given that the TEM-8 oral exam functions primarily as a language proficiency test, with interpreting serving as a key evaluative component, interpreting errors may serve as important indicators of broader challenges in learners’ language application. Consequently, English language instruction should not only aim to improve general linguistic and technical competence but also incorporate interpreting-focused pedagogical strategies that directly target the most frequent and impactful error types. Based on the study’s findings, several instructional strategies are recommended: shadowing exercises to enhance fluency and improve speech flow; note-taking practice to reduce information loss and strengthen message retention; and error-awareness tasks, including structured self-reflection and peer feedback on transcribed interpretations, to train learners to identify and self-correct recurring errors, particularly semantic deviations. Moreover, simulated interpreting tasks under time constraints can foster cognitive flexibility and support the development of emotional regulation skills, both of which are essential for managing the demands of real-time interpreting.

In addition, gender-responsive teaching approaches that account for individual differences in cognitive styles, affective factors, and learning strategies may further enhance the effectiveness of interpreter training. Integrating such strategies into routine classroom instruction can foster not only linguistic competence but also essential non-linguistic skills, including resilience, concentration, and adaptability, thereby better preparing learners for the demands of real-world interpreting contexts.

Nevertheless, the present study has certain limitations. While the PACCEL-S corpus provides a valuable and sizable dataset, uncontrollable variables, such as participants’ prior interpreting training experience and the duration or intensity of their training, may have influenced the quality of their performance. These factors, in turn, could affect the reliability and validity of the data analysis. It is important to recognize that such limitations are inherent to corpus-based studies of this nature.

Future research should adopt more rigorous participant selection criteria, exercise greater control over potential confounding variables, and refine data structures to enhance internal coherence. These improvements would strengthen the reliability, validity, and generalizability of findings, contributing to more robust conclusions and more precise theoretical development in interpreting studies.

## Note

According to Gile [1], phonological distortions are also considered a type of interpreting error. However, in the corpus used in the present study, the frequency of phonological errors is significantly lower than any other type of interpreting error, and therefore, they are not discussed further in the present study.

## Supporting information

S1 File Raw data. (XLSX)
